# P02.113. The benefits of yoga for women veterans with chronic low back pain

**DOI:** 10.1186/1472-6882-12-S1-P169

**Published:** 2012-06-12

**Authors:** E Groessl, K Weingart, N Johnson, S Baxi

**Affiliations:** 1 Veterans Affairs San Diego Healthcare System and University of California, San Diego, San Diego, USA; 2 Veterans Affairs San Diego Healthcare System, San Diego, USA

## Purpose

Chronic low back pain (CLBP) is prevalent among military veterans and often leads to functional limitations, psychological symptoms, lower quality of life, and higher health care costs. An increasing proportion of US veterans are women, and women veterans may have different healthcare needs than men veterans. The purpose of this study was to assess the impact of a yoga intervention on women and men with CLBP.

## Methods

Veterans Affairs (VA) patients with CLBP were referred by primary care providers to a clinical yoga program. Research participants completed a brief battery of questionnaires before their first yoga class and again 10 weeks later in a single group, pre-post study design. Questionnaires included measures of pain (Pain Severity Scale), depression (CESD-10), energy/fatigue, and health-related quality of life (SF-12). Yoga attendance and home practice of yoga were also measured. Repeated measures ANOVAs were used to analyze group differences over time while controlling for baseline differences.

## Results

The 53 participants who completed both assessments had a mean age of 53 years, were well educated, 41% non-White, 49% married, and had varying employment status. Women participants had significantly larger decreases in depression (p=.046) and pain “on average” (p=.050), but larger increases in energy (p=.034) and SF-12 Mental Health (p=.044) than men who participated. The groups did not differ significantly on yoga attendance or home practice of yoga.

## Conclusion

Our results suggest that women veterans may benefit more than men veterans from yoga interventions for chronic back pain. Conclusions are tentative because of the small sample size and quasi-experimental study design. A more rigorous study is being designed to answer these research questions more definitively.

